# Difference in 24-Hour Urine Composition between Diabetic and Non-Diabetic Adults without Nephrolithiasis

**DOI:** 10.1371/journal.pone.0150006

**Published:** 2016-02-23

**Authors:** Wei Zhu, Zanlin Mai, Jing Qin, Xiaolu Duan, Yang Liu, Zhijian Zhao, Jian Yuan, Shaw P. Wan, Guohua Zeng

**Affiliations:** 1 Department of Urology, Minimally Invasive Surgery Center, The First Affiliated Hospital of Guangzhou Medical University, Guangzhou, Guangdong, China; 2 Guangdong Key Laboratory of Urology, Guangzhou, Guangdong, China; Emory University, UNITED STATES

## Abstract

**Background:**

Diabetic patients are more likely to develop kidney stones than the general population. The underlying mechanisms for this disparity remain to be elucidated. Little is known about the relationship between urine composition and diabetes mellitus in non-stone-forming individuals. We sought to examine the differences in the 24-hour (24-h) urine composition between diabetic and non-diabetic adults who were not stone formers.

**Methods:**

A convenience sample of 538 individuals without a history of nephrolithiasis, gout, hyperparathyroidism, or gastroenteric diseases participated in this study. The 24-h urine profiles of 115 diabetic adults were compared with those of 423 non-diabetic adults. Diabetes was defined by self-reported physician diagnosis or medication use. All participants were non-stone formers confirmed by urinary tract ultrasonography. Participants provided a fasting blood sample and a single 24-h urine collection for stone risk analysis. Student’s t-test was used to compare mean urinary values. Linear regression models were adjusted for age, gender, body mass index, hypertension, fasting serum glucose, serum total cholesterol, estimated creatinine clearance rate and urinary factors.

**Results:**

Univariable analysis showed that the diabetic participants had significantly higher 24-h urine volumes and lower urine calcium and magnesium excretions than non-diabetic participants (all P < 0.05). After multivariate adjustment, no significant differences in 24-h urine composition were observed between diabetic and non-diabetic participants except for a slightly increased 24-h urine volume in diabetic participants (all P > 0.05). The main limitation of this study is that the convenience samples and self-reported data may have been sources of bias.

**Conclusion:**

Our data showed that there were no differences in 24-h urine composition between diabetic and non-diabetic adults who are not stone formers. The reason for it might be the improved glycemic control in diabetic individuals in our study. Therefore, a tighter glycemic control might reduce stone formation in diabetic adults.

## Introduction

Diabetes mellitus (DM) is associated with an increased risk of developing kidney stone.[1] Population-based cohort studies showed that diabetic patients are more likely to develop kidney stones than general population.[1] The underlying mechanisms for this disparity remain to be elucidated.

The 24-h urine collection has been the cornerstone for the evaluation of patients with nephrolithiasis.[2] Urine composition might be an important factor for the increase of stone formation in patients with DM.[3–4] Several studies had demonstrated that diabetic patients with kidney stones excreted more oxalate and uric acid, and had lower urine pH values than those of non-diabetic cohort.[3–4] However, these studies only included patients with history of nephrolithiasis, thus the results might not be applicable to the non-stone forming population. There was only one study that looked at the composition of 24-h urine specimens in diabetic patients and normal volunteers who were not stone formers. This study had very limited sample size.[5]

In the present study, our goal was to determine the differences in 24-h urine composition between the diabetic and non-diabetic adults using a broad base population. All participants in the present study had no history of kidney stones.

## Material and Methods

### Ethics Approval

This study was approved by the Ethics Committee of the First Affiliated Hospital of Guangzhou Medical University, China. In addition, written informed consents were obtained from all the participants.

### Study Population

We performed a clustered, stratified, and multi-stage screening for urolithiasis in six different cities of China between 2013 and 2014. The cities included Shanghai, Chongqing, Haerbing, Shaoyang, Lanzhou and Changzhi. All the participants were non-incentivized volunteers. After obtaining informed consent, each participant completed an initial questionnaire—a self-reporting system that collected information regarding his or her social and demographic status (e.g. gender and age), personal and family health history, lifestyle, and the use of vitamins and medications. Body measurements and laboratory analysis were carried out by trained professionals using standardized protocol. Urinary tract ultrasonography was performed for all participants. The estimated creatinine clearance rate (eCCr) was calculated by the Cockcroft-Gault equation:

$$eCCr\;\left(mL/min\right)=\frac{\left(140-\text{Age}\right)\times \text{Weight}\left(\text{kg}\right)}{\text{Cr}\left(\text{umol}/\text{L}\right)\times 0.818}\times \left(0.85\text{ if female}\right)$$

### Collections and Analysis of Urine

We sent a kit containing all the necessary supplies for the collection to each person. Urine samples were collected into clean polyethylene containers with toluene as preservative. One 24-h urine sample was collected from each individual. All participants stayed on their normal diet and fluid intake during the collection.

Urine oxalate and citrate were measured using ion exchange chromatography (Metrohm, Switzerland). Urine sodium, potassium, chloride, calcium, phosphate, and creatinine were determined by Unicel DxC 600 synchronic biochemical detecting system. Urine uric acid and magnesium were measured using Beckman coulter AU680 automatic biochemistry analyzer. Urine cystine was quantified using Thermo Scientific Microplate Reader. pH values were determined with a glass electrode in a calibrated pH meter (Mettler Toledo, Switzerland). All 24-h urinary analyses were performed in the Guangdong Key Laboratory of Urology according to standardized protocols. The urine was analyzed within 72 hours of collection.

### Ion Activity Product Risk Indices

Approximate estimates of ion activity products of calcium oxalate and calcium phosphate were expressed in terms of AP(CaOx) index_s_ and AP(CaP) index_s_ according to the formulas given in the following sections[6]. In the calculations, 24-h calcium, oxalate, citrate, magnesium, and phosphate were expressed in millimole and the volume in liters.

$$AP\left(CaOx\right)index_{\text{s}}=\frac{1.9{\text{*Calcium}}^{0.84}\text{*Oxalate}}{{\text{Citrate}}^{0.22}*Magnesium^{0.12}*1.5^{1.03}}$$

$$AP\left(CaP\right)index_{s}=\frac{2.7\text{*}10^{-3}*Calcium^{1.07}*Phospate^{0.70}*{\left(7.0-4.5\right)}^{6.8}}{{\text{Citrate}}^{0.20}*1.5^{1.31}}$$

### Subject Selection

Diabetic individuals were identified by self-reporting. The diagnosis was further corroborated by the use of anti-hyperglycemic medication. To avoid incomplete collections, we only accepted participants whose 24-h urine creatinine values were greater than 600 mg for women and 800 mg for men. Subjects were also excluded from this study if they met the following criteria:

- Had self-reported gastroenteric diseases;
- Had self-reported gout;
- Had self-reported primary or secondary hyperparathyroidism;
- Had self-reported history of previous urinary stones;
- Had urinary stones confirmed by ultrasonography;
- Had high serum creatine level (>133 umol/L);
- Had taken thiazide, allopurinol, vitamin supplements, calcitonin, potassium citrate or calcium supplements within the past two weeks;
- Participants who did not report DM but had fasting serum glucose ≥ 7.0 mmol/L.

**Fig 1:**
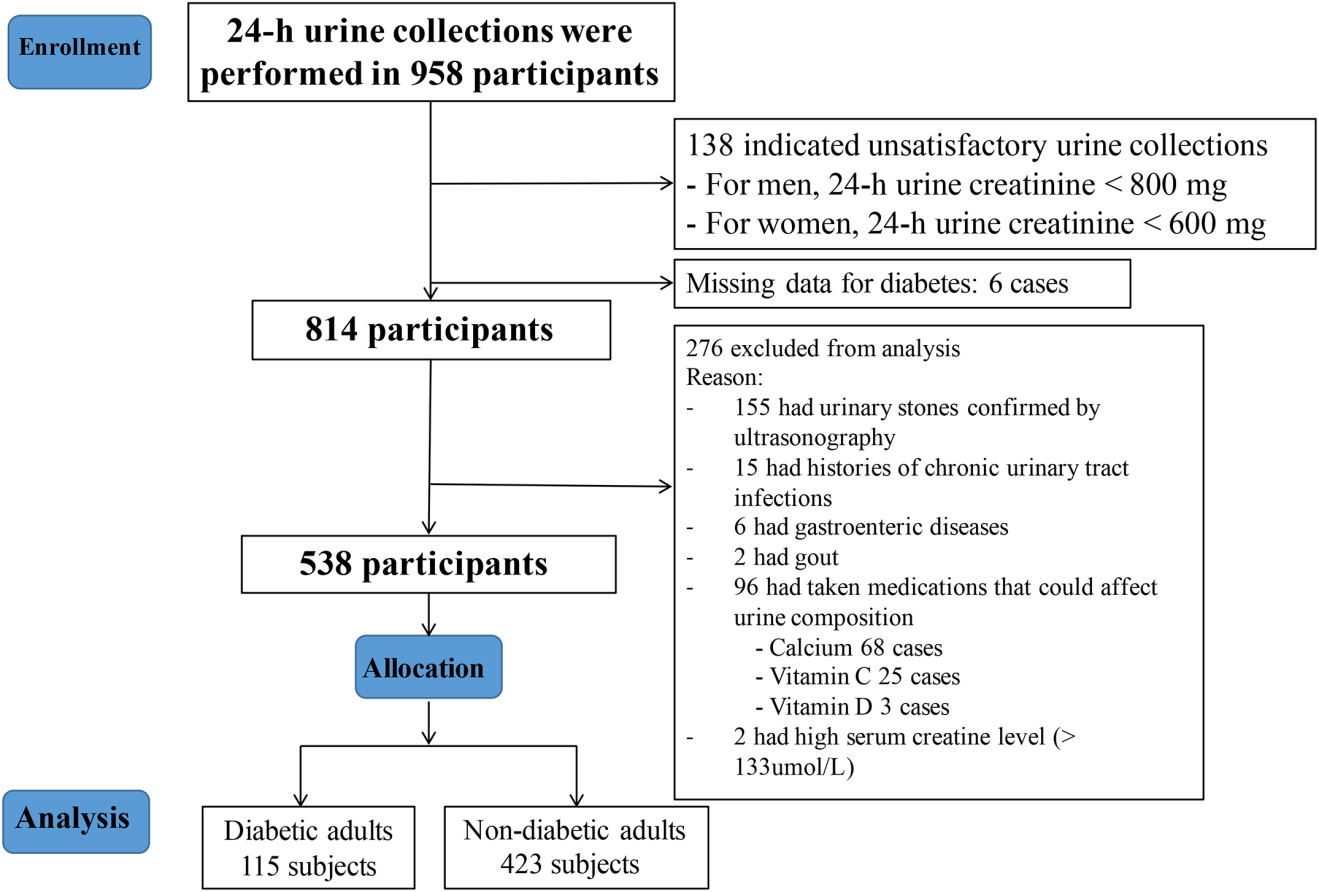
CONSORT flow diagram. Subject selection process.

The number of subjects who were excluded based on these criteria was 420 (333 of them had DM and 87 did not, p = 0.803). Total of 538 subjects met our criteria and were included in this study. 115 of them had DM and 423 did not. (Fig 1)

### Statistical Analysis

Comparisons of the baseline characteristics between the diabetic and non-diabetic participants were performed using the Student’s t-test for continuous variables and chi-squared for categorical variables. Univariable comparisons of urinary components between diabetic and non-diabetic adults were accomplished using the Student’s t-test. Multivariate linear regression was adjusted for possible confounders, including age, gender, body mass index (BMI), hypertension, fasting glucose, total cholesterol and eCCr. We did not transform the non-normally distributed urinary factors because the residuals in the regression analyses approximated for normal distribution and our sample size was relatively large. Two-tailed P value < 0.05 was considered to be statistically significant. The data were analyzed using the SPSS 13.0 software.

## Results

Characteristics of the diabetic and non-diabetic participants were shown in Table 1. Diabetic participants were older, had higher BMI and lower eCCr value. They also had higher levels of fasting glucose and total cholesterol. In addition, they were more likely to have hypertension than the non-diabetic participants.

**Table 1:** Mean demographic in diabetic and non-diabetic adults^a^.

Characteristic	Diabetic adults (n = 115)	Non-diabetic adults (n = 423)	p Value
Age (year)	63.22 ± 10.58	49.99 ± 13.39	<0.001
BMI (kg/m^2^)	24.84 ± 4.40	23.77 ± 3.20	0.004
Gender, Male (n [%])	41 (35.65%)	163 (38.53)	0.572
Hypertension (n [%])	11 (9.57%)	6 (1.42%)	<0.001
Fasting glucose (mmol/L)	5.29 ± 1.24	5.02 ± 0.82	0.025
Total cholesterol (mmol/L)	4.98 ± 1.00	4.54 ± 1.10	<0.001
Triglyceride (mmol/L)	1.46 ± 0.79	1.36 ± 1.01	0.316
Hemoglobin (g/L)	135.94 ± 16.45	136.93 ± 16.73	0.571
Serum creatine (umol/L)	67.45 ± 15.98	73.80 ± 18.06	<0.001
eCCr mL/min	80.80 ± 28.06	86.85 ± 26.60	0.033

^a^ Data are means ± SD except where noted.

Univariable analysis showed that the diabetic participants had significantly higher 24-h urine volumes (Table 2). Diabetic participants also had significantly lower urine calcium and magnesium excretions than non-diabetic participants.

**Table 2:** Mean 24-h urinary excretion in diabetic and non-diabetic adults.

Parameter	Diabetic adults	Non-diabetic adults	p Value
Calcium (mmol)	3.63 ± 2.50	4.17 ± 2.08	0.020
Oxalate (mmol)	0.28 ± 0.17	0.26 ± 0.15	0.177
Citrate (mmol)	2.22 ± 1.65	2.28 ± 1.37	0.690
Uric acid (mmol)	3.10 ± 1.04	2.99 ± 1.04	0.289
Sodium (mmol)	169 ± 75.26	176 ± 76.03	0.429
Potassium (mmol)	46 ± 17.69	43 ± 19.58	0.077
Magnesium (mmol)	3.37 ± 1.72	3.79 ± 1.61	0.015
Phosphate (mmol)	17.18 ± 6.31	16.20 ± 6.36	0.141
Chloride (mmol)	166 ± 73.61	172 ± 73.22	0.386
Creatinine (mmol)	8.95 ± 2.75	9.40 ± 2.68	0.111
Cystine (mmol)	0.31 ± 0.26	0.33 ± 0.32	0.369
Volume (ml)	1522.70 ± 637.84	1371.89 ± 627.39	0.023
pH	6.34 ± 0.79	6.23 ± 0.64	0.122
AP (CaOx) Index_s_	0.74 ± 0.66	0.77 ± 0.58	0.628
AP (CaP) Index_s_	21.54 ± 19.41	23.91 ± 16.65	0.192

The results of multivariate linear regression analysis comparing the differences in urinary factors between the diabetic and non-diabetic participants were shown in Table 3. After adjustment for age, gender, BMI, hypertension, fasting glucose, total cholesterol and eCCr, there were no differences noted in all urinary factors between diabetic and non-diabetic participants except for a slightly increased 24-h urine volume in diabetic participants. The results were not changed materially without adjustment for BMI and fasting glucose. Additional adjustment for urinary volume, urinary pH and all other urinary factors also did not change the results.

**Table 3:** Multivariate adjusted differences in 24-h urinary excretion between diabetic and non-diabetic adults.

Parameter	Difference	95% CI	P Value
Calcium (mmol)	-0.283	(-0.765 to 0.198)	0.248
Oxalate (mmol)	0.031	(-0.005 to 0.066)	0.087
Citrate (mmol)	-0.113	(-0.432 to 0.110)	0.206
Uric acid (mmol)	0.196	(-0.036 to 0.429)	0.098
Sodium (mmol)	0.788	(-16.045 to 17.620)	0.927
Potassium (mmol)	1.837	(-2.490 to 6.164)	0.405
Magnesium (mmol)	-0.142	(-0.493 to 0.208)	0.425
Phosphate (mmol)	0.939	(-0.476 to 2.354)	0.193
Chloride (mmol)	0.742	(-15.439 to 16.923)	0.928
Creatinine (mmol)	-0.072	(-0.576 to 0.432)	0.779
Cystine (mmol)	0.012	(-0.198 to 0.081)	0.735
Volume (ml)	145.660	(1.689 to 289.631)	0.047
pH	0.128	(-0.027 to 0.283)	0.105
AP(CaOx) Index_s_	0.033	(-0.103 to 0.168)	0.635
AP(CaP) Index_s_	-0.728	(-4.524 to 3.067)	0.706

Non-diabetic adults group is the referent. Differences are adjusted for age, gender, BMI, hypertension, fasting glucose, total cholesterol and eCCr. CI, confidence interval

## Discussion

DM was reported to be associated with an increased risk of kidney stone formation.[1] Recently attention had turned to the urinary characteristics that might contribute to this increased risk of stone formation. Hartman et al [4] found that urine pH was significantly lower in the urolithiasis patients with DM as compared to patients without DM. They also found increased oxalate and uric acid in patients with DM. Eisner et al[3] reported that stone forming patients with type II DM excreted significantly greater amount of urinary oxalate and had significantly lower urine pH values than those without DM. Fram et al.[7] also found that kidney stone patients with diabetes had lower urine pH, calcium and phosphate when compared with non-diabetic stone former. All these reports did not include individuals who were not stone formers; therefore, their results might not be applicable to the diabetic patients without kidney stones. Up to now there were only limited data focusing the relationship between urine composition and DM in the non-stone-forming individuals.

Uric acid stones were known to be more prevalent in diabetic patients.[8–9] The relation between DM and uric acid nephrolithiasis was attributed to the effects of insulin resistance on urine pH and the renal handing of ammonium and calcium.[8, 10] Insulin was known to simulate the synthesis of ammonia in the renal tubule that mediates ammonium excretion in urine. In addition, insulin has been shown to enhance parallel uric acid and sodium reabsorption in the proximal convoluted tubule, which in turn resulted in hyperuricemia and decreased uric acid and sodium excretion.[11] This is a very important mechanism for preventing the urinary pH to decrease to very low levels. Insulin resistance may decrease the ammonium production by competitive mechanisms, direct lipo-toxicity and interference with the transport mechanisms in the tubular cell membrane.[12–13] In the idiopathic calcium stone formers, insulin resistance is also associated with lower urine citrate excretions, likely to be due to the same mechanism of urine acidification.[14] In a small cohorts study, Cameron et al[5] compared the urine composition of 24 patients with DM who were not stone-formers to 59 normal volunteers. They found that DM patients had a significantly higher serum glucose levels (7.57 vs 5.19 mmol/L) and lower 24-h urine pH values than normal volunteers (5.66 vs 6.07).

In the present study, univariable and multivariable analyses showed that diabetic adults did not have significantly lower urine pH values than non-diabetic adults. The reason for this might be the improved glycemic control in our diabetic participants. In our study, the mean fasting glucose level in diabetic participants was in the normal range (5.29 ± 1.24 mmol/L) though it was higher than that of non-diabetic participants. Torricelli et al[11] observed that there was an inverse correlation between HbA1c level and urine pH in patients with type-II diabetes. Glycemic control might have a protective effect for low urine pH and consequently reduce the risk of stone formation.

Several studies have suggested that poor glycemic control could be an independent risk factor for kidney stones.[10, 15] Kebaya et al[15] in a cross-sectional study with 2171 patients reported that patients with fasting plasma glucose concentrations ≥ 7.0 mmol/L had an odds ratio of 1.83 for forming kidney stones as compared to patients with fasting plasma glucose concentrations < 5.52 mmol/L. Their result was similar to those reported by Weinberg et al(10) in the cross-sectional study of 12,110 participants in the National Health and Nutrition Examination Survey. Weinberg et al.[10] reported that HbA1c and a history of insulin use were strongly associated with self-reported stone disease. Fram et al.[7] observed that diabetics with worse glycemic control as measured by HbA1c had changes in their excretion of citrate, creatinine, and total urine volume independent of urine pH. Insulin use was associated with alterations in urinary parameters expected to be protective for stone disease.

In addition to the lower urine pH, several studies had reported that the diabetic stone formers had greater urine oxalate, sulphate, chloride, and uric acid excretions, but less citrate excretion than the non-diabetic stone formers.[3–5] Furthermore, Nagasaka et al[16] reported an increase in the urinary calcium and phosphorus excretion in diabetic patients. Mandel et al.[17] showed that diabetes was associated with higher urinary citrate excretion in non-stone formers. In the present study, we had not observed any difference in urinary compositions between the diabetic and non-diabetic non-stone-forming individuals. There were two possible reasons to explain why our results were not consistent with the others. One might be the better glycemic control in the diabetic individuals in our study. Hyperglycemia and its resultant of glycosuria had been implicated to impair the renal handling of calcium and phosphorus. Because proximal tubular reabsorption of most organic molecules occurs through sodium cotransport, excess filtered glucose may compete with citrate for reabsorption through this pathway.[17] These dysfunctions could be corrected by improved glycemic control.[10, 16] The second reason might be the influence of urinary stones on the composition of 24-h urine in the other reports in the literature. Laube et al[18] reported that the presence of urinary stones could affect the composition of 24-urine. They indicated that ideally evaluation of the patient’s metabolic status should be undertaken only after total stone removal. If there was any stone material actually present in the patient’s urinary tract, it was necessary to take them into account. In our study, all the participants were confirmed non-stone formers and so this situation did not arise.

To our knowledge, this was the largest broad based cohorts study to compare 24-h urine characteristics between the non-stone forming persons with DM to those without DM. The strengths of this study included that our subjects were selected from 6 different cities in China and all were examined with ultrasonography. Furthermore, the availability of laboratory data on the fasting glucose, total cholesterol and triglyceride allowed us to be more perceptive in analyzing the differences in 24-h urine composition between diabetic and non-diabetic adults. In addition, we also ruled out several diseases, such as gout and primary or secondary hyperparathyroidism, which might influence the urinary composition.

This study has its limitations. The most important one was the fact that DMs were self-reported. Nevertheless, we did validate the self-reported DM with fasting serum glucose. Another limitation of the study was the use of convenience sampling rather than randomized sampling, since this may have introduced a sampling bias. We also could not adequately adjust for multiple dietary factors. Many pertinent data were not available in the present study. Other potential sources of error included over- and under-collection of 24-h urine samples. Individuals with poorly controlled diabetes have larger urine volumes and are more likely to have more urine than fits in a standard collection jug. They void more often compared to non-diabetics, so they have more opportunities to mishandle urine causing it to not be included.[19] Finally, all the participants in this study were ethnic Chinese, the results might not be applicable to the other racial groups.

## Conclusion

Our data showed that there were no differences in the 24-h urine composition between the diabetic and non-diabetic adults without kidney stones. The reason for it might be the improved glycemic control in diabetic individuals in our study. Therefore, a tighter glycemic control might reduce stone formation in diabetic adults. Other mechanism that could trigger the increased risk of stone formation in the diabetic patients should be further investigated.

## Supporting Information

S1 Checklist This file is the STROBE Statement—Checklist of items that should be included in reports of cross-sectional studies. (DOC)

S1 Data This file is the dataset of our study. (XLS)
